# Subnanomolar indazole-5-carboxamide inhibitors of monoamine oxidase B (MAO-B) continued: indications of iron binding, experimental evidence for optimised solubility and brain penetration

**DOI:** 10.1080/14756366.2017.1344980

**Published:** 2017-07-20

**Authors:** Nikolay T. Tzvetkov, Liudmil Antonov

**Affiliations:** a NTZ Lab Ltd., Sofia, Bulgaria;; b Bulgarian Academy of Sciences, Institute of Organic Chemistry, Centre of Phytochemistry, Sofia, Bulgaria

**Keywords:** Alzheimer’s disease, free energy calculations, iron chelators, MAO inhibitors, Parkinson’s disease

## Abstract

Pharmacological and physicochemical studies of *N*-unsubstituted indazole-5-carboxamides (subclass I) and their structurally optimised N1-methylated analogues (subclass II), initially developed as drug and radioligand candidates for the treatment and diagnosis of Parkinson’s disease (PD), are presented. The compounds are highly brain permeable, selective, reversible, and competitive monoamine oxidase B (MAO-B) inhibitors with improved water-solubility and subnanomolar potency (pIC_50 _>8.8). Using a well-validated, combined X-ray/modelling technology platform, we performed a semi-quantitative analysis of the binding modes of all compounds and investigated the role of the indazole N1 position for their MAO-B inhibitory activity. Moreover, compounds NTZ-1006, 1032, and 1441 were investigated for their ability to bind Fe^2+^ and Fe^3+^ ions using UV-visible spectroscopy.

## Introduction

Alzheimer’s disease (AD) and Parkinson’s disease (PD) are the most prevalent, aging-related neurodegenerative disorders of the central nervous system (CNS), currently affecting over 8% of individuals aged ≥65 years worldwide. Despite their differences in pathogenesis and symptoms, AD and PD share common underlying features such as chronic, irreversible, and progressive neuronal degradation in the human brain caused by complex pathophysiological processes, including oxidative stress, neuro-inflammation, excitotoxicity, mitochondrial dysfunction, and proteolytic stress^1–3^. The currently approved anti-AD and anti-PD drugs have an impact on several symptoms in different disease stages and improve quality of life for patients, but do not stop the disease progression nor exhibit a neurorestorative effect^4^^,^^5^.

**Figure 1.**
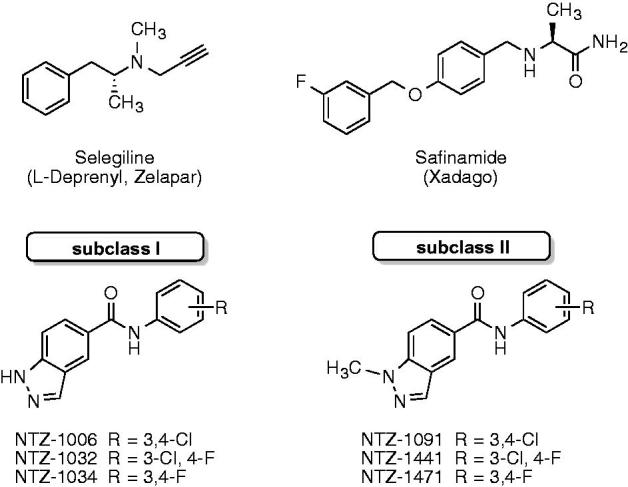
Structures of selective MAO-B inhibitors.

Monoamine oxidases (MAOs, EC 1.4.3.4) are mitochondrial flavoenzymes that play a key role in the metabolism of monoaminergic neurotransmitters. Two isoforms of MAOs are present in mammals, MAO-A and MAO-B, differing by their distribution in the human brain, substrate preference, and selectivity to different inhibitors^6^^,^^7^. The expression levels and activity of MAO-B, but not of MAO-A, increase with aging, leading to an enhanced production of reactive oxygen species (ROS)^8^^,^^9^. Some studies suggest that high-level accumulation of iron may also contribute to higher ROS production and neurotoxicity^1^^,^^10^. Overproduction of ROS is associated with oxidative stress and neuronal death in patients with AD and PD^8–10^. Therefore, selective inhibition of MAO-B is a well-established approach for treatment of PD^11–13^. For example, the irreversible MAO-B inhibitor selegiline and the reversible inhibitor safinamide are approved for the treatment of PD^11–13^ (Figure 1). Recently we have discovered a series of selective MAO-B or dual MAO-A/B inhibitors with nanomolar potency^14^; several compounds of structurally related *N*-unsubstituted indazole-5-carboxamides^15^ (subclass I) and N1-methylated indazole-5-carboxamides^16^ (subclass II) were identified as best-in-class MAO-B inhibitors (Figure 1). In contrast to previously reported 5-carboxamidoindole derivatives^17^, the herein presented indazole-5-carboxamides contain a reversed carboxamide linker and an additional indazole N2 atom leading to very favourably water interactions and, therefore, to notably affinity increase^16^.

In this work we report on the pharmacological, physicochemical, and photophysical evaluation of structurally optimised N1-methyl indazole-5-carboxamides as brain penetrant, reversible, and competitive MAO-B inhibitors with improved physicochemical parameters. Such compounds are considered for further exploration *in vivo* as anti-PD and anti-AD therapeutic and radioligand candidates.

## Experimental

Details of the experimental procedures for the biological and physicochemical assays are provided in the *Supporting Information*.

### MAO inhibition assay

The evaluation of human MAO inhibitory activity of the indazole-5-carboxamides and reference inhibitors was assessed by a fluorescence-based assay measuring their effects on the production of hydrogen peroxide (H_2_O_2_) from *p*-tyramine, a common substrate for both MAO isoforms, using microsomes of baculovirus-infected insert cells (BTI-TN-5B1–4) as sources for the human recombinant MAO isoforms and the Amplex Red MAO assay kit (Molecular Probes Inc., Eugene, OR)^15^. The standard drugs clorgyline (MAO-A) and selegiline (MAO-B) were used as positive controls. The kinetics of the hMAO-B enzyme reaction were determined in the presence of different *p*-tyramine concentrations (0.12–1.0 mM). In our experiments, hMAO-B displayed a Michaelis constant (*K*_m_) of 118.8 ± 1.23 μM and a maximal velocity (*V*_max_) of 40.4 ± 1.13 nmol *p*-tyramine/min/mg protein (*n* = 3)^16^. Determined *in vitro* inhibitory potencies (IC_50_ values), selectivity towards hMAO-A and hMAO-B (expressed as the selectivity index, SI), together with the respective *K*_i_ values at hMAO-B (calculated from the mean IC_50_) for all compounds and reference inhibitors are reported in Table 1. In addition, we estimated the affinities (*K*_i HYDE_ ranges) obtained from the best scored compounds’ docking solutions (see molecular modelling studies) and the thermodynamic parameters of binding to hMAO-B using a novel free energy approximation “HYDE” embedded in SeeSAR v.5.5 software^18^.

**Table 1.** MAO inhibitory activity and HYDE estimated affinities against hMAO-B.

	IC_50_ (nM)^a^					Binding affinity (kJ/mol)^e^		
Compound	hMAO-A	hMAO-B	SI^b^	*K*_i_ (nM)^c^	*K*_i HYDE_ (nM)^d^	ΔH	−TΔS	ΔG
**NTZ-1006**	>10,000	0.59 ± 0.09	>16959	0.26 ± 0.04	2–185	−38.0	−6.2	−44.2
**NTZ-1032**	>10,000	0.68 ± 0.04	14706	0.30 ± 0.02	4–400	−38.0	−4.2	−42.2
**NTZ-1034**	>10,000	1.59 ± 0.16	>6289	0.70 ± 0.07	5–464	−38.0	−3.8	−41.8
**NTZ-1091**	>10,000	0.39 ± 0.05	25,641	0.17 ± 0.02	0–37	−38.0	−9.9	−47.9
**NTZ-1441**	≥10,000	0.66 ± 0.06	≥15,151	0.29 ± 0.03	1–103	−38.0	−7.5	−45.5
**NTZ-1471**	>10,000	1.52 ± 0.18	6579	0.67 ± 0.08	1–112	−38.0	−7.3	−45.3
**Selegiline**	1700^f^	6.59 ± 1.09^f^	258	2.91 ± 0.48	8–757	0.0	−41.4	−41.4
**Safinamide**	45,000^f^	5.18 ± 0.04^f^	5000^f^	2.29 ± 0.02	2–187	−21.8	−22.5	−44.3

a The values are the mean ± SEM (*n* = 3).

b SI: Selectivity Index = IC_50_ (hMAO-A)/IC_50_ (hMAO-B).

c The *K*_i_ values were calculated from the experimentally measured IC_50_ hMAO-B values according to the equation by Cheng and Prusoff^24^: *K*_i _=IC_50_ (1 + [S]/*K*_m_) with substrate concentration of *p*-tyramine [S] = 150 μM and Michaelis constant *K*_m _=118.8 μM.

d *K*_i HYDE_: estimated HYDE *K*_i_ range values from the compounds’ best docking conformations within the human MAO-B (PDB: 2V5Z^20^).

e HYDE estimated thermodynamic binding values; ΔG: Gibbs free energy = ΔH –TΔS; ΔH: sum of interactions (enthalpy); −TΔS: sum of desolvation terms (entropy).

f Data from Ref.^15^.

### Molecular modelling studies

Docking studies, estimation, and visualisations of hMAO-B binding affinities were carried out according to a recently developed modelling workflow using LeadIT v.2.1.8^19^ and SeeSAR v.5.5 software packages^18^ (both from BioSolveIT GmbH, Sankt Augustin, Germany) with HYDE visual affinity assessment^16^.

#### Ligand and protein preparation

Ligand input structures were taken from the respective single molecule crystal X-ray structures of compounds NTZ-1006, 1034, 1091, and 1041 and used without further preparation^16^. The crystal structure of the hMAO-B enzyme in complex with safinamide (PDB: 2V5Z^20^) was obtained from the Protein Data Bank (PDB).

**Figure 2.**
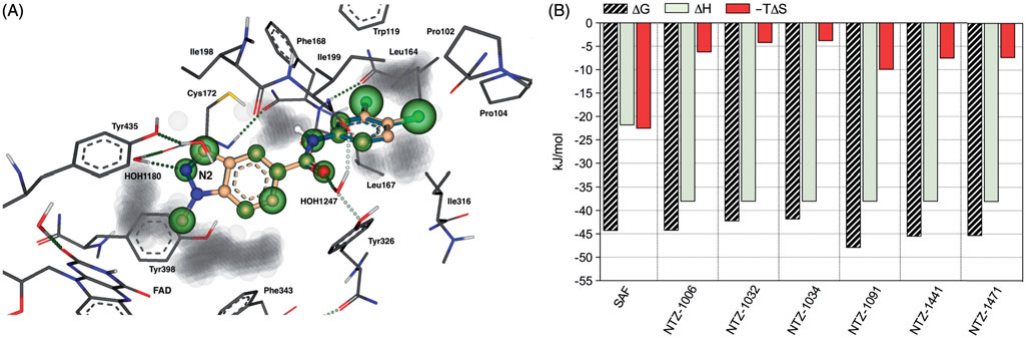
(A) SeeSAR visualisation of the binding of NTZ-1006 (blue) and NTZ-1091 (off-white) overlaid onto the crystal structure of the hMAO-B-safinamide complex (PDB: 2V5Z). HYDE visual affinity assessment: green = favourable, red = unfavourable and non-coloured = not relevant for affinity. (B) Bar diagrams representing a semi-quantitative decomposition of enthalpic (ΔH: sum of interactions) and entropic part (−TΔS: sum of desolvation terms) for all heavy atoms of the Gibbs free energy (ΔG, kJ/mol) of tested compounds in comparison to the reference MAO-B inhibitor safinamide (SAF).

#### Pose generation and docking

Docking experiments were performed using the FlexX docking module in LeadIT v.2.1.8^19^ with the same procedure as reported previously^16^. LeadIT has accurately reproduced the experimental binding mode of safinamide in 2V5Z and yielded very plausible and well-scored poses on high ranks for all docked compounds discussed in this and earlier works. The ranking of the generated poses corresponds always with the measured binding affinity (IC_50_ and *K*_i_ values) of the tested compounds and their best docking conformations. For some compounds, the SeeSAR-integrated docking engine was applied to generate a maximum of 10 poses as output.

#### Hyde scoring and visualisation

The top 23 LeadIT poses were loaded into SeeSAR for post-scoring with HYDE visual affinity assessment. Compounds NTZ-1032 and NTZ-1471 were built using the SeeSAR-integrated editor. SeeSAR visualises the estimated free energy of binding (ΔG) using “coronas” that range from dark red (very unfavourable) to large dark green spheres (very favourable for affinity)^21^. The selection of the best poses was based on their visual HYDE scores while also considering a statistics-based torsional analysis^22^. The software enables an interactive assessment of torsions and energies (in kJ/mol) including the desolvation (dehydration, −TΔS) and enthalpic (interaction, ΔH) contributions to binding for both protein and ligand. Furthermore, SeeSAR can visualise and quantitatively report the energy contributions for all heavy atoms (with a united atom approach for bounded H-atoms) and allows a semi-quantitative estimation of the thermodynamic profile for all tested compounds and safinamide in the co-crystal structure 2V5Z (Figure 2).

**Figure 3.**
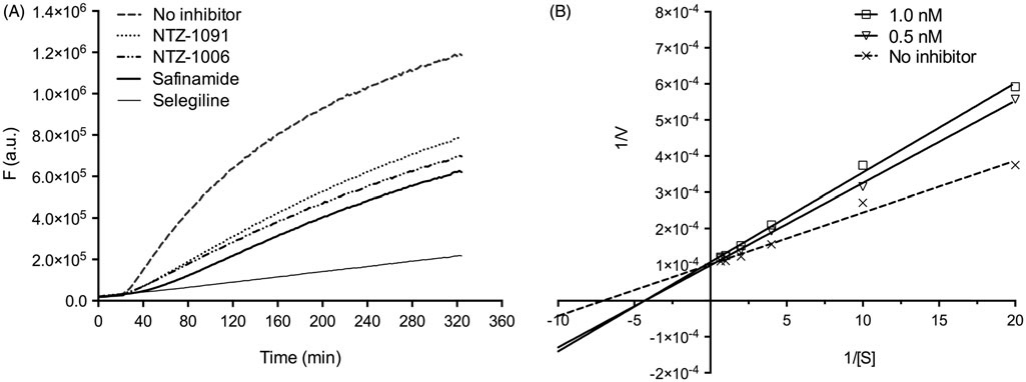
(A) Reactivation of recombinant human MAO-B enzyme by irreversible inhibitor selegiline (30 nM), reversible inhibitor safinamide (50 nM), and representative compounds NTZ-1006 (1.0 nM) and NTZ-1091 (1.0 nM). (B) Lineweaver–Burk plot of the inhibition of hMAO-B enzyme in the absence (no inhibitor) and in the presence of different concentrations (0.5 and 1.0 nM) of NTZ-1091. The reciprocal hMAO-B inhibitory activity of the compound was plotted versus the reciprocal substrate concentration. 1/*V*: 1/velocity of reaction [1/(nmol *p*-tyramine/min/mg protein)]; 1/[S]: 1/substrate concentration (1/mM *p*-tyramine).

### Ligand ADME

Kinetic solubility of compound NTZ-1034 and octanol-water distribution coefficients (logD) of NTZ-1034, 1091, 1441, and 1471 were determined using a protocol described earlier^16^. Ligand physicochemical estimations were carried out using the StarDrop model runner algorithms in SeeSAR v.5.5^18^. The *in silico* physicochemical, drug-like, and *in vitro* ADME properties of investigated and reference MAO-B inhibitors are summarised in Table 2.

**Table 2.** Physicochemical, drug-like, and *in vitro* ADME properties of investigated and reference MAO-B inhibitors.

Compound	MW	pIC_50_	Stability	*t*PSA^a^	%ABS	HBA/D^a^	logBB^a^	logS_7.4_	logD_7.4_	LLE
**NTZ-1006**	306.15	9.23	Stable	57.78	89.07	4/1	0.065	n.d. (−4.56^a^)	n.d. (3.73^a^)	5.50
**NTZ-1032**	289.04	9.17	Stable	57.78	89.07	4/2	0.030	n.d. (−4.48^a^)	n.d. (3.00^a^)	6.17
**NTZ-1034**	273.24	8.80	Stable	57.78	89.07	4/2	−0.002	−4.22 (−4.61^a^)	2.04 (2.40^a^)	6.76
**NTZ-1091**	320.17	9.41	Stable	46.92	92.81	4/2	0.246	−4.75 (−4.82^a^)	3.24 (3.76^a^)	6.17
**NTZ-1441**	303.06	9.18	Stable	46.92	92.81	4/2	0.216	−3.87 (−4.76^a^)	2.19 (3.15^a^)	6.99
**NTZ-1471**	287.27	8.82	Stable	46.92	92.81	4/2	0.187	−3.67 (−4.81^a^)	n.d. (2.64^a^)	6.18
**SEL**	187.29	8.18	–	3.24	107.88	4/1	0.570	n.d. (−4.10^a^)	n.d. (2.35^a^)	5.83
**SAF**	302.35	8.29	–	64.68	86.67	4/1	−0.083	n.d. (−4.06^a^)	n.d. (2.89^a^)	5.40
^b,^^c^**CNS+**	≤400	>8	–	<70	≥60	≤7/≤3	≥–1	≥ −5.0	1–4	>5

MW: molecular weight; pIC_50_ at human MAO-B; Stability: chemical stability of compounds in 10 mM phosphate buffer at pH 7.4; *t*PSA: topological surface area (in Å^2^); %ABS: % of absorption = 109–0.345×*t*PSA (Ref.^29^); HBA/D: number of hydrogen bond acceptors/donors; logBB: blood-brain partition coefficient (lit. 31); logS: solubility (in mol/L) at pH 7.4 in 60 mM phosphate buffer or pure water at rt; logD: distribution coefficient at pH 7.4 in 60 mM phosphate buffer at rt; LLE: ligand-lipophilicity efficiency = pIC50–logD (Ref.^30^); n.d.: not determined; SEL: Selegiline; SAF: Safinamide.

a Calculated values using the StarDrop module in SeeSAR 5.5, 2017^18^.

b Ref.^25–27^.

c CNS+: required ranges for compound penetration to the central nervous system (CNS).

### Solubility studies

The solubility of selected compounds in pure water (Purelab flex) and 50% methanol was measured by a combined HPLC-UV/ESI-MS analysis using different measurement techniques as described in the Supporting Information.

### Parallel artificial membrane permeability assay (PAMPA)

**Figure 4.**
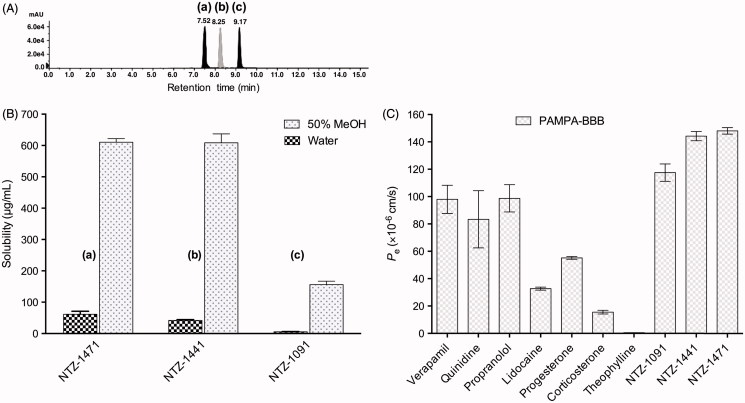
(A) HPLC chromatogram of a standard 1.0 mM equimolar mixture of compounds NTZ-1471 (a, ret. time 7.52 min), NTZ-1441 (b, ret. time 8.25 min) and NTZ-1091 (c, ret. time 9.17 min) in methanol (MeOH). Conditions: 40% methanol mobile phase, 10 μL sample injection volume, and 250 μL/min flow rate. (B) Mean solubility of the compounds in pure water and 50% methanol measured by LC/ESI-MS method. (C) Distribution of BBB mean permeability of standard drugs and subclass II compounds measured in the PAMPA assay.

The PAMPA permeability of selected compounds and standard drugs across the artificial blood-brain barrier (BBB) lipid membrane was performed by measuring the UV–visible absorbance of compounds in both donor and acceptor compartments, using the PAMPA Explorer kit (Pion Inc., Billerica, MA). Measurements were performed at room temperature with incubation for 4 h under stirring. The *P*_e_ and –log*P*_e_ values were processed using the PAMPA Explorer software v.3.8 (Pion) and the data are presented as the mean ± SD (Table 3, Figure 4). The permeability data obtained for the standard drugs were used for method validation (see Table S2, Supporting Information). Among these, verapamil and theophylline were used as references for compounds with high and low permeability, respectively. The plot of the experimental permeability versus the reported values of these commercial drugs gave a good linear correlation with –log*P*_e_ (exp) = 1.007 –log*P*_e_ (rep) + 0.1334 and R^2^ = 0.9046 (see Figure S3). Based on this equation and considering the well-established limit for BBB permeation (*P*_e_ > 4.0 × 10^−6 ^cm/s)^23^, compounds NTZ-1091, 1441, and 1471 can be classified as highly BBB permeable, indicating that these should cross the BBB and reach the CNS therapeutic targets (CNS+).

**Table 3.** Solubility and PAMPA-BBB data for selected compounds.

	Solubility in water^a^		Solubility in 50% methanol^a^		PAMPA-BBB^a,^^b^	
Compound	μg/mL	μM	μg/mL	mM	*P*_e_ (×10^−6^ cm/s)	−log*P*_e_
**NTZ-1091**	5.62 ± 0.17	17.6 ± 0.5	156 ± 19	0.49 ± 0.06	117.5 ± 6.4	3.91
**NTZ-1441**	42.1 ± 2.3	136 ± 5	609 ± 49	2.04 ± 0.18	144.2 ± 3.4	3.84
**NTZ-1471**	61.6 ± 9.6	215 ± 33	610 ± 26	2.12 ± 0.09	148.1 ± 2.4	3.83

a The values are the mean ± SD (*n* ≥ 3).

b PAMPA blood-brain barrier (BBB) values were determined under stirring at the pH value of the Prisma HT buffer (Pion).

### Metal binding studies

Binding ability of compounds with the Fe^2+^ and Fe^3+^ ions was determined by measuring the UV–vis absorbance spectra of the respective compound in the absence and in the presence either of FeCl_3_ or FeSO_4_. Deferiprone (DFP) was used as a standard chelating agent.

## Results and discussion

All compounds under investigation were prepared by amide coupling reactions of differently substituted amines with *N*-unsubstituted carboxylic acids or their N1-methylated analogues for the formation of *N*-unsubstituted indazole-5-caroxamides (designated subclass I, compounds NTZ-1006, 1032, and 1034) or N1-methylated indazole-5-carboxamides (subclass II, NTZ-1091, 1441, and 1471), respectively^14^. Therefore, the main goal of this study was to investigate the role of the indazole N1 position for MAO-A/B selectivity and inhibitory activity as well the effects of this structural modification on different physicochemical and biophysical properties within indazole-5-carboxamides of subclass I and II. In general, all tested compounds are highly potent and selective inhibitors of hMAO-B isoenzyme with IC_50_ values in the subnanomolar or even picomolar range (Table 1). The 3,4-dichlorophenyl-substituted compounds NTZ-1006 (hMAO-B, IC_50_ = 0.56 nM; SI >16,000) and NTZ-1091 (hMAO-B, IC_50_ = 0.39 nM; SI >25,000) were found to be the most potent and selective MAO-B inhibitors in both series, being ∼9-fold and >13-fold more potent against hMAO-B than the approved drug safinamide. Compared to the irreversible MAO-B inhibitor selegiline, compounds NTZ-1006 and NTZ-1091 display a > 11- and ∼17-fold increase in inhibitory activity against hMAO-B, respectively. The IC_50_ values and SI slightly decrease within both subclasses with decreasing the lipophilic character (Cl *vs.* F) of the compounds as follows: 3,4-*di*-Cl (NTZ-1006, 1091) > 3-Cl, 4-F (NTZ-1032, 1441) > 3,4-*di*-F (NTZ-1034, 1471). The introduction of a methyl substituent at the indazole N1 position of subclass I compounds led to a slight increase in inhibitory potency and selectivity of subclass II compounds toward hMAO-B, indicating that small lipophilic substituents at the indazole N1 atom are well-tolerated by hMAO-B. None of the tested compounds exhibited inhibitory activity against the hMAO-A isoform at the initial tested concentration of 10 μM.

In order to validate the biological testing results and to gain insight into the binding thermodynamics of the investigated compounds to hMAO-B, we computed their binding modes with LeadIT^19^ using the X-ray co-crystal structure of hMAO-B with the reference inhibitor safinamide (PDB: 2V5Z)^20^. Next, the free energy of bindings were calculated and visualised with the software SeeSAR^19^ applying a novel, well-validated computational free energy approximation “HYDE” as embedded in SeeSAR^16^. Finally, we compared the results computed in SeeSAR for the best docking poses of our compounds with their biological data (*K*_i_ values). The *K*_i_ values were calculated from the corresponding mean IC_50_ values using the Cheng–Prusoff equation^24^ and the hMAO-B enzyme kinetic parameters. In general, there is a good overall agreement between the experimental (*K*_i_ values in pM range) and the computed affinity ranges, e.g. HYDE scores (*K*_i HYDE_ ranges in low nM to pM).

Furthermore, we performed a semi-quantification of the enthalpic (sum of H-bond interactions, ΔH) and entropic (sum of dehydration effects: −TΔS) parts of all non-hydrogen atoms contributing to the Gibbs free energy (ΔG) of binding to hMAO-B using HYDE scoring in SeeSAR^16^. The results obtained from the HYDE analysis of all tested compounds and reference inhibitors reproduced very well their hMAO-B activities. The most potent compounds NTZ-1006 and NTZ-1091 in both subclasses, showed the highest HYDE estimated thermodynamic binding affinity of –44.2 and –47.9 kJ/mol, respectively (Table 1).

The binding modes and estimated affinities strongly suggest that compounds NTZ-1006 and 1091 occupy the same substrate cavity region within the binding pocket of hMAO-B, provided that both ligands are very compatible with the active site and do not covalently bind to flavin adenine dinucleotide (FAD) cofactor (Figure 2(A)). The active region consists of FAD, the main residues Tyr398, and 435, and the water molecule 1180. The indazole N2 atom and the carboxamide linker of NTZ-1006 and 1091 play an essential role to their conformation and binding affinity, interacting favourably with the water molecules HOH1180 and 1247, respectively.

The thermodynamic profiles computed by HYDE (enthalpy-entropy effects) of all investigated compounds to hMAO-B showed similar, predominant enthalpic contribution of −38 kJ/mol and favourable entropic terms ranging between −3.8 (NTZ-1034) and −9.9 kJ/mol (NTZ-1091) to the total binding energy (Figure 2(B)). In contrast, safinamide showed almost equal, favourable contributions of the enthalpic (interaction) and entropic (desolvation) effects to the binding energy.

In our research program we are particularly interested in further development of reversible MAO inhibitors due to the considerable advantages compared to irreversible inhibition of MAO^14–16^. Reactivation experiments were performed to evaluate the mode of interaction (reversible or irreversible) of the most potent compounds NTZ-1006 and NTZ-1091 in both series with the active site of the hMAO-B (Figure 3(A). The inhibition of reactivated hMAO-B was measured at concentrations that correspond to compounds IC_80_ values versus the increased concentration of the substrate *p*-tyramine after pre-incubation for 22 min. In the experiments with the inhibitors NTZ-1006 and 1091, an elevated fluorescence can be detected after increasing the concentration of *p*-tyramine, similar to that observed for the reversible MAO-B inhibitor safinamide, suggesting that NTZ-1006 and 1091 are reversible inhibitors of hMAO-B. In the experiments with the irreversible inhibitor selegiline, the residual enzyme activity was not considerably improved. The reversibility studies are in agreement with the results obtained from the docking experiments for the reversible mode of inhibition of NTZ-1006 and 1091.

To further investigate the type of enzyme inhibition of the most potent compound NTZ-1091 with the binding site of hMAO-B, Michaelis–Menten kinetic experiments were performed at different concentrations of *p*-tyramine in the absence (no inhibitor) and in the presence of two different concentrations of the representative compound NTZ-1091 (Figure 3(B)). The Lineweaver–Burk plots of the data for NTZ-1091 were linear and intersected at the Y-axis with the plot for the uninhibited hMAO-B enzyme. The Michaelis constant (*K*_m_) increase with higher inhibitor concentration while the maximal velocity (*V*_max_) remains constant at different concentrations of NTZ-1091. The obtained results clearly indicate that the *N*-unsubstituted (subclass I compounds) and their N1-methylated analogues (subclass II) are reversible MAO-B inhibitors with competitive mode of inhibition.

A good hydrophilicity-lipophilicity balance is a specific requirement for the development of oral bioavailable CNS active drugs. Therefore, we evaluated the physicochemical, drug-like, and relevant ADME (absorption, distribution, metabolism, and excretion) properties of the tested compounds and the reference MAO-B inhibitors selegiline and safinamide (Table 2). The chemical stability of all tested compounds was confirmed by long-terms studies, which is a necessary precondition for their further development as drug and radioligand candidates. Overall, the physicochemical properties for the investigated compounds are in the suggested strict limits for drug-likeness of CNS active (CNS+) drugs (MW ≤400, HBA <7, HBD <3, and *t*PSA <70 Å^2^)^16^^,^^25–27^. Oral bioavailability is multifactorial property, primary driven by the gastrointestinal (GI) absorption (expressed as %ABS). Furthermore, the topological polar surface area (*t*PSA) is a key descriptor that correlates with passive transport through membranes (GI and BBB) and, therefore, used for calculation of %ABS^28^. The *t*PSA values for all compounds are in the range of 46.9–57.8 Å^2^ and, consequently, the %ABS ranges from 89.1 to 92.8%, indicating that all investigated compounds are expected to be by orally bioavailable (%ABS ≥60%) and classified as good brain penetrable (*t*PSA ≤60 Å^2^) CNS drug candidates^25^^,^^29^^,^^30^. To further predict the BBB permeability of compounds, we also calculated their blood (plasma)-brain coefficients (logBB)^31^. The logBB values for all tested compounds are in the suggested limit for BBB permeable drugs (logBB>–1)^25^, being even higher than the one of the approved drug safinamide (logBB = −0.083).

Solubility and lipophilicity are key ADME parameters that effect pharmacokinetics and pharmacodynamics of drugs. The measured and calculated water-solubility (expressed as logS_7.4_) and distribution coefficients (logD_7.4_) for the tested compounds are in the ideal range (logS_7.4_ ≥ −5.0, logD_7.4_ = 1–4)^31^, suggesting a good solubility-lipophilicity balance that is suitable for GI absorption by passive membrane permeation after oral administration^26^.

The ligand-lipophilicity efficiency (LLE), a drug-like multiparameter that combines lipophilicity and *in vitro* potency (pIC_50_ or p*K*_i_) of a drug candidate, for all tested compounds is in the range between 5 and 7 that is required for their further *in vivo* evaluation^29^^,^^31^. The investigated compounds show higher LLE scores than those of reference inhibitors selegiline and safinamide.

The investigated indazole-5-carboxamides show optimal physicochemical, drug-like, and ADME properties. Compounds NTZ-1441 and 1471 can be highlighted because of their improved drug-likeness (MW = 287–303, %ABS = 92.8%; logS_7.4 _>–4.0, logD_7.4_ < 3, LLE >6–7) and predicted brain permeability combined with optimal hMAO-B inhibitory activity (pIC_50 _>8.0). Subsequently, we evaluated subclass II compounds for their BBB permeability using PAMPA-BBB assay (Table 3). Comparison of solubility data with BBB permeability for these compounds confirms their predicted high brain penetration in combination with good water-solubility. As shown in Figure 4, the solubility and BBB permeability of the representative compounds increased in the rank order as follows: 3,4-di-Cl-Ph (NTZ-1091) < 3-Cl-4-F-Ph (NTZ-1441) ≈ 3,4-di-F-Ph (NTZ-1471). Moreover, all three compounds exhibit considerably higher BBB permeability that those observed for the standard drugs used in the experiments (Figure 4(C)).

**Figure 5.**
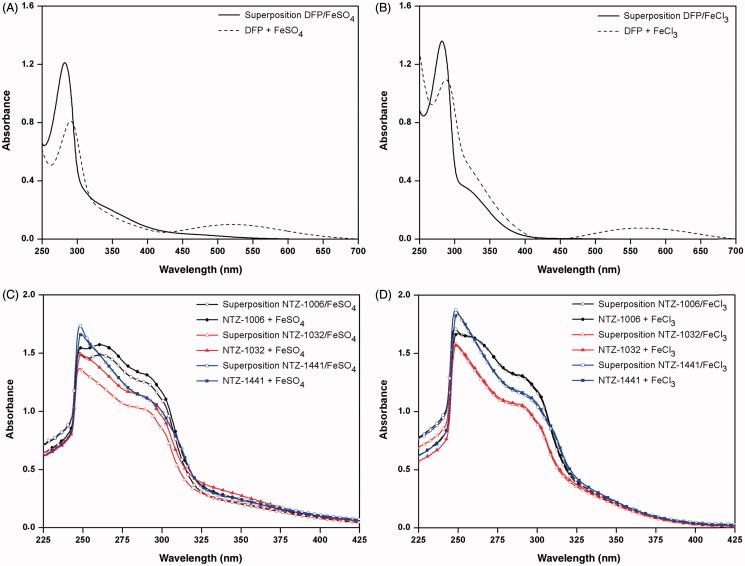
UV–visible studies of Fe^2+^ or Fe^3+^ chelating ability of deferiprone (DFP) in the absence or presence of 10 mM FeSO_4_ (A) and FeCl_3_ (B) and compounds NTZ-1006 (black line), NTZ-1032 (red line), and NTZ-1441 (blue line) before and after addition of 40 μL of 10 mM FeSO_4_ (C), or FeCl_3_ (D). Superposition UV–visible spectra were obtained by measurement of absorbance of compound alone (50 μM) and FeSO_4_ or FeCl_3_ without compound (both 130 μM) in DMSO 50%.

Considering the growing interest in the development of multiefficient drugs including iron chelating agents for the treatment of AD^1^^,^^3^, we have preliminary investigated compounds NTZ-1006, 1032, and 1441 for their ability to bind di- and trivalent iron ions (Figure 5). Therefore, we measured the UV–visible absorbance of these compounds in the absence and in the presence either of Fe^2+^ or Fe^3+^ and compared this behaviour with that obtained for the reference drug DFP. As expected, DFP exhibited higher chelating affinity against Fe^3+^ ions. However, its UV–visible spectra were significantly changed by the addition either of FeSO_4_ or FeCl_3_ resulting in an increase in the specific profile at 521 or 565 nm, respectively (Figure 5(A) and (B)). The N2-methylated compound NTZ-1441 did not have the ability to bind iron cations. Compounds NTZ-1006 and 1032 show similar chelating ability. The maximum absorbance of both compounds was not modified by the addition of Fe^3+^, whereas a slight increase of the maximum absorbance was observed when FeSO_4_ was added to the respective solutions of NTZ-1006 or 1032, indicating that both compounds selectively interact with Fe^2+^ ions.

## Conclusion

In conclusion, newly discovered *N*-unsubstituted indazole-5-caroxamide derivatives (subclass I compounds) and their N1-methyl analogues (subclass II compounds) are subnanomolar potent, reversible and competitive MAO-B inhibitors with the ability to selectively bind Fe^2+^ ions, as measured for compounds NTZ-1006 and 1032. The reversible mode of binding within the binding pocket of the human MAO-B enzyme was investigated using time-dependent studies; it was confirmed by applying a novel modelling and visualisation technique. In general, all investigated compounds exhibit suitable drug-like properties required for CNS active drugs (LLE >5.5, logD = 2–4, MW 287–320 Da). Optimisation of the indazole moiety in subclass I compounds has resulted in the discovery of compounds NTZ-1441 and NTZ-1471, highly BBB permeable, water-soluble MAO-B inhibitors that combine both well-balanced physicochemical and *in vitro* ADME properties with remarkable hMAO-B activity and selectivity against the hMAO-A isoform. The compounds all together appear as promising drug and radioligand candidates for the therapy and diagnosis of PD, AD, and other CNS diseases.

## Supplementary Material

IENZ_1344980_Supplementary_Material.pdf

## References

[1] Santos MA, Chand K, Chaves S. Recent progress in multifunctional metal chelators as potential drugs for Alzheimer´s disease. Coord Chem Rev 2016;327–328:287–303.

[2] Meredith GE, Totterdell S, Beales M, et al Impaired glutamate homeostasis and programmed cell death in a chronic MPTP mouse model of Parkinson’s disease. Exp Neurol 2009;219:334–40. 19523952 10.1016/j.expneurol.2009.06.005 PMC2728791

[3] Belaidi AA, Bush AI. Iron neurochemistry in Alzheimer’s disease and Parkinson’s disease: targets for therapeutics. J Neurochem 2016;139(Suppl I):179–97. 10.1111/jnc.13425 26545340

[4] Dunkel P, Chai CL, Sperlágh B, et al Clinical utility of neuroprotective agents in neurodegenerative disease: current status of drug development for Alzhemer’s, Parkinson’s and Huntington´s diseases, and amyotrophic lateral sclerosis. Exp Opin Investig Drugs 2012;21:1267–308. 10.1517/13543784.2012.703178 22741814

[5] Jankovic J, Poewe W Therapies in Parkinson’s disease. Curr Opin Neurol 2012;25:433–47. 22691758 10.1097/WCO.0b013e3283542fc2

[6] Tong J, Meyer JH, Furukawa Y, et al Distribution of monoamine oxidase proteins in human brain: implication for brain imaging studies. J Cereb Blood Flow Metab 2013;33:863–71. 23403377 10.1038/jcbfm.2013.19 PMC3677103

[7] Shih JC, Chen K, Ridd MJ. Monoamine oxidase: from genes to behavior. Annu Rev Neurosci 1999;22:197–217. 10202537 10.1146/annurev.neuro.22.1.197 PMC2844879

[8] Nicotra A, Pierucci F, Parvez H, et al Monoamine oxidase expression during development and aging. NeuroToxicol 2004;25:155–65. 10.1016/S0161-813X(03)00095-0 14697890

[9] Youdim MBH, Lavie I. Selective MAO-A and MAO-B inhibitors, radical scavengers and nitric oxide synthase inhibitors in Parkinson’s disease. Life Sci 1994;55:2077–82. 7527888 10.1016/0024-3205(94)00388-2

[10] Wang Y, Wang H, Chen H-Z. AChE inhibition-based multi-target-directed ligands, a novel pharmacological approach for the symptomatic and disease-modifying therapy of Alzheimer’s disease. Curr Neuropharmacol 2016;14:364–75. 26786145 10.2174/1570159X14666160119094820 PMC4876592

[11] Fowler JS, Logan J, Volkow ND, et al Evidence that formulations of the selective MAO-B inhibitor selegiline, which bypass first-pass metabolism, also inhibit MAO-A in the human brain. Neuropsychopharmacology 2015;40:650–7. 25249059 10.1038/npp.2014.214 PMC4289953

[12] Kumar B, Sheetal Mantha AK, et al Recent developments on the structure-activity relationship studies of MAO inhibitors and their role in different neurological disorders. RSC Adv 2016;6:42660–83.

[13] Fabbri M, Rosa MM, Abreu D, et al Clinical pharmacology review of safinamide for the treatment of Parkinson’s disease. Neurodegener Dis Manag 2015;5:481–96. 26587996 10.2217/nmt.15.46

[14] Tzvetkov NT. Substituted indazole or indole derivatives as in vitro MAO-B inhibitors. Patent WO2014/107771, 2014.

[15] Tzvetkov NT, Hinz S, Gastreich M, et al Indazole- and indole-5-carboxamides: selective and reversible monoamine oxidase B inhibitors with subnanomolar potency. J Med Chem 2014;57:6679–703. 24955776 10.1021/jm500729a

[16] Tzvetkov NT, Stammler H-G, Neumann B, et al Crystal structures, binding interactions, and ADME evaluation of brain penetrant N-substituted indazole-5-carboxamides as subnanomolar, selective monoamine oxidase B and dual MAO-A/B inhibitors. Eur J Med Chem 2017;127:470–92. 28107736 10.1016/j.ejmech.2017.01.011

[17] Carradori S, Silvestri R. New frontiers in selective human MAO-B inhibitors. J Med Chem 2015;58:6717–32. 25915162 10.1021/jm501690r

[18] SeeSAR v.5.5, BioSolveIT GmbH, Sankt Augustin, Germany 2017 Available from: http://www.biosolveit.de/SeeSAR.

[19] LeadIT v.2.1.9, BiosolveIT GmbH, Sankt Augustin, Germany 2016 Available from: http://www.biosolveit.de/LeadIT.

[20] Binda C, Wang J, Pisani I, et al Structures of human monoamine oxidase B complexes with selective noncovalent inhibitors: safinamide and coumarin analogs. J Med Chem 2007;50:5848–52. 17915852 10.1021/jm070677y

[21] Schneider N, Hindle S, Lange G, et al Substantial improvements in large-scale redocking and screening using the novel HYDE scoring function. J Comput Aided Mol Des 2012;26:701–23. 22203423 10.1007/s10822-011-9531-0

[22] Schärfer C, Schulz-Gasch T, Ehrlich HC, et al Torsion angle preferences in druglike chemical space: a comprehensive guide . J Med Chem 2013;56:2016–28. 23379567 10.1021/jm3016816

[23] Di L, Kerns EH, Fan K, et al High throughput artificial membrane permeability assay for blood-brain barrier. Eur J Med Chem 2003;38:223–32. 12667689 10.1016/s0223-5234(03)00012-6

[24] Cheng YC, Prusoff WH. Relationships between the inhibition constant (K_i_) and the concentration of inhibitor which causes 50% inhibition (IC50) of an enzymatic reaction. Biochem Pharmacol 1973;22:3099–108. 4202581 10.1016/0006-2952(73)90196-2

[25] Clark DE Rapid calculation of polar molecular surface area and its application to the prediction of transport phenomena. 1. Prediction of intestinal absorption. J Pharm Sci 1999;88:807–14. 10430547 10.1021/js9804011

[26] Wager TT, Hou X, Verhoest PR, Villalobos A. Moving beyond rules: the development of a central nervous system multiparameter optimization (CNS MPO) approach to enhance alignment of drug-like properties. ACS Chem Neurosci 2010;1:435–49. 22778837 10.1021/cn100008c PMC3368654

[27] Hitchcock SA, Pennington LD. Structure-brain exposure relationships. J Med Chem 2006;49:7559–83. 17181137 10.1021/jm060642i

[28] Ahsan MJ, Samy JG, Khalilullah H, et al Molecular properties prediction and synthesis of novel 1,3,4-oxadiazole analogues as potent antimicrobial and antitubercular agents. Bioorg Med Chem Lett 2011;21:7246–50. 22071303 10.1016/j.bmcl.2011.10.057

[29] Abad-Zapatero C. Ligand efficiency indices for effective drug discovery. Expert Opin Drug Discov 2007;2:469–88. 23484756 10.1517/17460441.2.4.469

[30] Vilar S, Chakrabarti M, Costanzi S. Prediction of passive blood-brain partitioning: straightforward and effective classification models based on in silico derived physicochemical descriptors. J Mol Graph Model 2010;28:899–903. 20427217 10.1016/j.jmgm.2010.03.010 PMC2873098

[31] Johnson TW, Dress KR, Edwards M. Using the Golden Triangle to optimize clearance and oral absorption. Bioorg Med Chem Lett 2009;19:5560–4. 19720530 10.1016/j.bmcl.2009.08.045

