# Supplementary material for: Subnanomolar indazole-5-carboxamide inhibitors of monoamine oxidase B (MAO-B) continued: indications of iron binding, experimental evidence for optimised solubility and brain penetration
Source: J Enzyme Inhib Med Chem. 2017 Jul 20;32(1):960–7. doi: 10.1080/14756366.2017.1344980 (PMC6445166; doi:10.1080/14756366.2017.1344980)
Supplement: IENZ_1344980_Supplementary_Material.pdf [file IENZ_A_1344980_SM4009.pdf]

Supporting Information

Subnanomolar indazole-5-carboxamide inhibitors of monoamine oxidase B (MAO-B) continued: indications of iron binding, experimental evidence for optimized solubility and brain penetration

Nikolay T. Tzvetkov[a,*], Liudmil Antonov[b]

[a] *NTZ Lab Ltd., Krasno selo 198, Sofia 1618, Bulgaria*

[b] *Bulgarian Academy of Sciences, Institute of Organic Chemistry, Centre of Phytochemistry, Acad. G. Bonchev Str., Bl. 9, Sofia 1113, Bulgaria*

*Corresponding author: Dr. Nikolay T. Tzvetkov, Tel: +49-179-528-4358 E-mail: ntzvetkov@ntzlab.com

Bioassays

MAO inhibition assay

The inhibitory activity of the compounds in Table 1 on both human MAO isoforms was assessed by a continuous fluorescence-based assay[1] with modification of previously described protocols[2]. Test samples for the MAO assays (1.0 mM final concentration in DMSO) were prepared by a dilution with DMSO. The following stock solutions were used: test compounds (10 mM) in DMSO, reference MAO inhibitors (0.5 mM), *p*-tyramine (100 mM), and resorufin sodium salt (2.0 mM) in deionized water. Commercially available recombinant human MAO-A and MAO-B enzymes (Sigma-Aldrich, M7316 and M7441) expressed in baculovirus-infected BTI insect cells were used for determination of hMAO-A and hMAO-B inhibition, respectively. *p*-Tyramine was used as a substrate (150 µM final concentration). Inhibition assays were conducted at room temperature in 96-well plates (200 µL final volume) by using the commercial MAO assay Amplex Red kit (pH 7.4). The irreversible MAO-A inhibitor clorgyline and the irreversible MAO-B inhibitor selegiline (each in a final concentration of a 1.0 µM) were used to determine the specific non-MAO-A and non-MAO-B enzyme activity, respectively, and subtracted from the total enzyme activity measured. The concentration-response curves of clorgyline (MAO-A) and selegiline (MAO-B) served as positive controls. A sample with DMSO (2.0 µL) was used as a negative control. Fluorescence measurements were performed in triplicate for 45 min at room temperature using a microplate fluorescence reader PHERA star FSX (BMG Labtech) with an excitation at 544 nm and an emission at 590 nm. Inhibition of hMAO isoenzymes was detected with two initial concentrations (10 and 0.1 µM) prior determination of full inhibition of the test compound at 1.0 mM final concentration. The IC_{50} values were determined from the respective dose-response inhibition curves and are expressed as mean IC_{50} value ± standard error of the mean (SEM) of at least three independent experiments.

Reversibility of MAO-B

Evaluation of the type of binding of inhibitors NTZ-1006 and NTZ-1091 to human MAO-B enzyme was performed by time-dependent inhibition experiments with modification of previously reported protocols[2,3]. The irreversible selegiline (30 nM final concentration) and the reversible safinamide (50 nM final concentration) MAO-B inhibitors were used as references. Indazole-based compounds (1.0 nM final concentration) as well as reference inhibitors were studied at their IC_{80} values without pre-incubation of the hMAO-B enzyme/inhibitor mixtures. Control experiments without inhibitors were run for each compound. The enzyme reaction was started by adding 10 μM of the substrate *p*-tyramine and the enzymatic activity of the tested compounds was measured for 22 min. Then, the substrate concentration was increased to 1.0 mM final concentration of *p*-tyramine and the enzyme reactivation was monitored by fluorescence measurements for 5 h.

MAO-B kinetic experiments

The mechanism of hMAO-B inhibition of the representative compound NTZ-1091 was evaluated in substrate-dependent kinetic experiments[2,3]. The catalytic rates of hMAO-B were measured at different concentration of *p*-tyramine substrate (0.05, 0.1, 0.25, 0.5, 1.0, and 1.5 mM) in the absence (basal sample) and in the presence of two different concentrations (0.5 and 1.0 nM) of NTZ-1091. The results are presented as double reciprocal Lineweaver-Burk plots (see Figure 3B). The measurements were conducted in duplicate using the same hMAO-B assay conditions as described for the determination of IC_{50} values.

Physicochemical Assays

Apparatus and conditions

The purity and solubility of the compounds was determined by an analytical HPLC-UV method using an LC-MS instrument (Applied Biosystems API 2000 LC/ESI-MS; HPLC Agilent 1100 binary pump system) coupled with a diode array detector (DAD) with an UV absorption in the range from 220 to 400 nm. Mass spectra were recorded on an API 2000 mass spectrometer equipped with an electrospray ion source (ESI) by using triple ion quadrupole (TIC) with an alternating full scan detection in positive (+Q) and/or negative (-Q) mode. As mobile phases for the different analytical LC methods were used methanol (Sigma-Aldrich, Chromasolv LC-MS grade), ammonium acetate (2.0 mM) and water Purelab flex.

Chemical stability

500 µL of a 10 mM compound stock solution in DMSO, ACN or MeOH was added to a 2.0 mL HPLC glass vial containing 500 µL phosphate buffer (10 mM, pH 7.4) and sonicated for 60 sec. The sample was shaken at 37 °C for 2 hours and then left for 60 days at room temperature under light exposure. The chemical stability of compounds was checked by UV-visible spectroscopy (Figure S1).

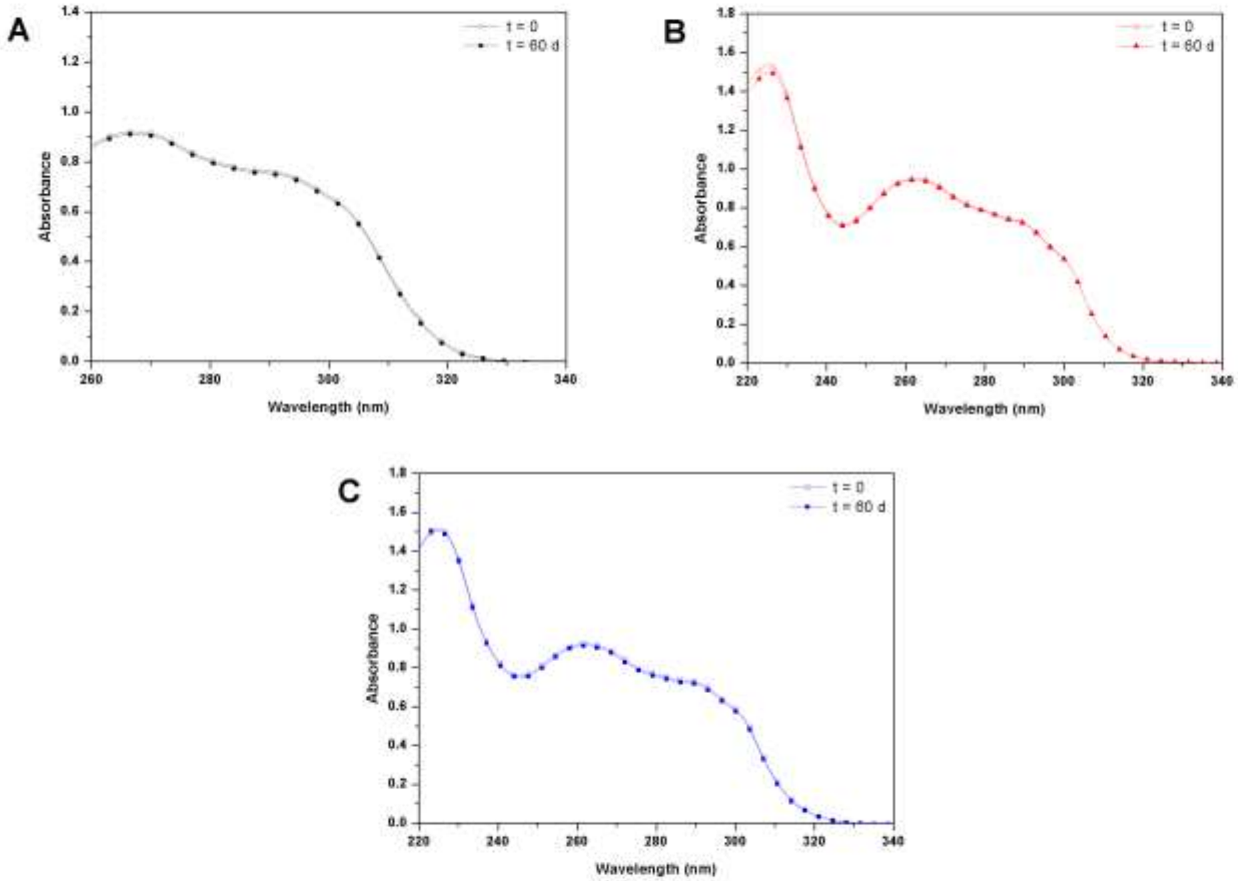

Figure S1. UV-vis spectra of chemical stability studies of NTZ-1006 in DMSO (A), acetonitrile (B), and methanol (C) measured at t = 0 and after 60 days at room temperature.

Solubility studies

Solubility of compounds NTZ-1091, 1441, and 1471 in pure water and 50% methanol was determined by combined HPLC-UV/ESI-MS analysis with modification of previously published protocols[4,5]. Solid samples of compounds (2–4 mg) were weighed and serial diluted in 1.0 mL either of water (Purelab flex) or water-methanol (1:1). A precisely weighed sample of the respective compound in methanol (1.0 ± 0.05 mg/mL) was used as a reference. A blank solution without detectable analyte was used for instrument calibration for each experiment. The suspensions were mixed on a vortex for 5 min, sonicated for 1 h, and then shaken over night at rt. After that, the samples were left for 1 h at rt and then filtered through a 0.45 µm filter. Aliquots (10 µL) of each sample were injected into the HPLC/ESI-MS system (Applied

Biosystems API 2000 ESI-MS, HPLC Agilent 1100 binary pump system) equipped with a Phenomenex Luna C18 reverse phase column and subsequently measured by chromatographic procedure (DAD detection) with mass conformation (MS scan in positive and negative mode). The mass scan method was adapted for each tested compound depending on its limit of detection (LOD) and physicochemical properties (Table S1, Figure S2). The solubility of the compounds was calculated by Analyst software v. 1.5.1 (AB Sciex Pte. Ltd.) using one point calibration after subtraction the absorbance of the blank solution from the total analyte peak area measured for each sample in the experiments. To verify the results, an equimolar mixture (each in a concentration equal to 1.0 mg/mL) of the compounds in methanol was prepared and measured under the same conditions as described above (see Figure 4A). All experiments were performed in triplicate with at least three analytical runs for each sample.

Solubility data of compounds NTZ-1091, NTZ-1441, and NTZ-1471

The solubility data for tested compounds, obtained from the different analytical techniques, are summarized in Table S1.

Table S1. Solubility data for selected compounds.

**Compd.**	**Method**[a]	**Solubility in 50% methanol**[b]			**Solubility in water**[b]		
		**S (µg/mL)**	**S (mM)**	**logS**	**S_w (µg/mL)**	**S_w (µM)**	**logS_w**
**NTZ-1091** MW 319.028	**1**	105.3 ± 5.05	0.30 ± 0.03	–3.48 ± 0.02	0.75 ± 0.00	2.36 ± 0.00	–5.63 ± 0.00
	**2**	156.0 ± 19.0	0.49 ± 0.06	–3.35 ± 0.04	5.62 ± 0.17	17.6 ± 0.46	–4.75 ± 0.01
**NTZ-1441** MW 303.057	**1**	662.9 ± 3.58	2.19 ± 0.01	–2.66 ± 0.00	10.6 ± 0.94	34.9 ± 3.11	–4.46 ± 0.04
	**2**	608.8 ± 48.7	2.04 ± 0.18	–2.69 ± 0.04	42.1 ± 2.25	136.1 ± 5.16	–3.87 ± 0.02
**NTZ-1471** MW 287.087	**1**	630.7 ± 16.05	2.18 ± 0.07	–2.66 ± 0.014	10.4 ± 0.62	36.3 ± 2.33	–4.41 ± 0.03
	**2**	610.3 ± 26.2	2.12 ± 0.09	–2.67 ± 0.02	61.6 ± 9.56	214.5 ± 33.3	–3.67 ± 0.07

[a] Method of detection: HPLC-UV method 1 (DAD), HPLC/ESI-MS method 2 (+Q/–Q scan: +MI+H^+/–MI–H^-). [b] The values are the mean ± SD ($n \geq 3$).

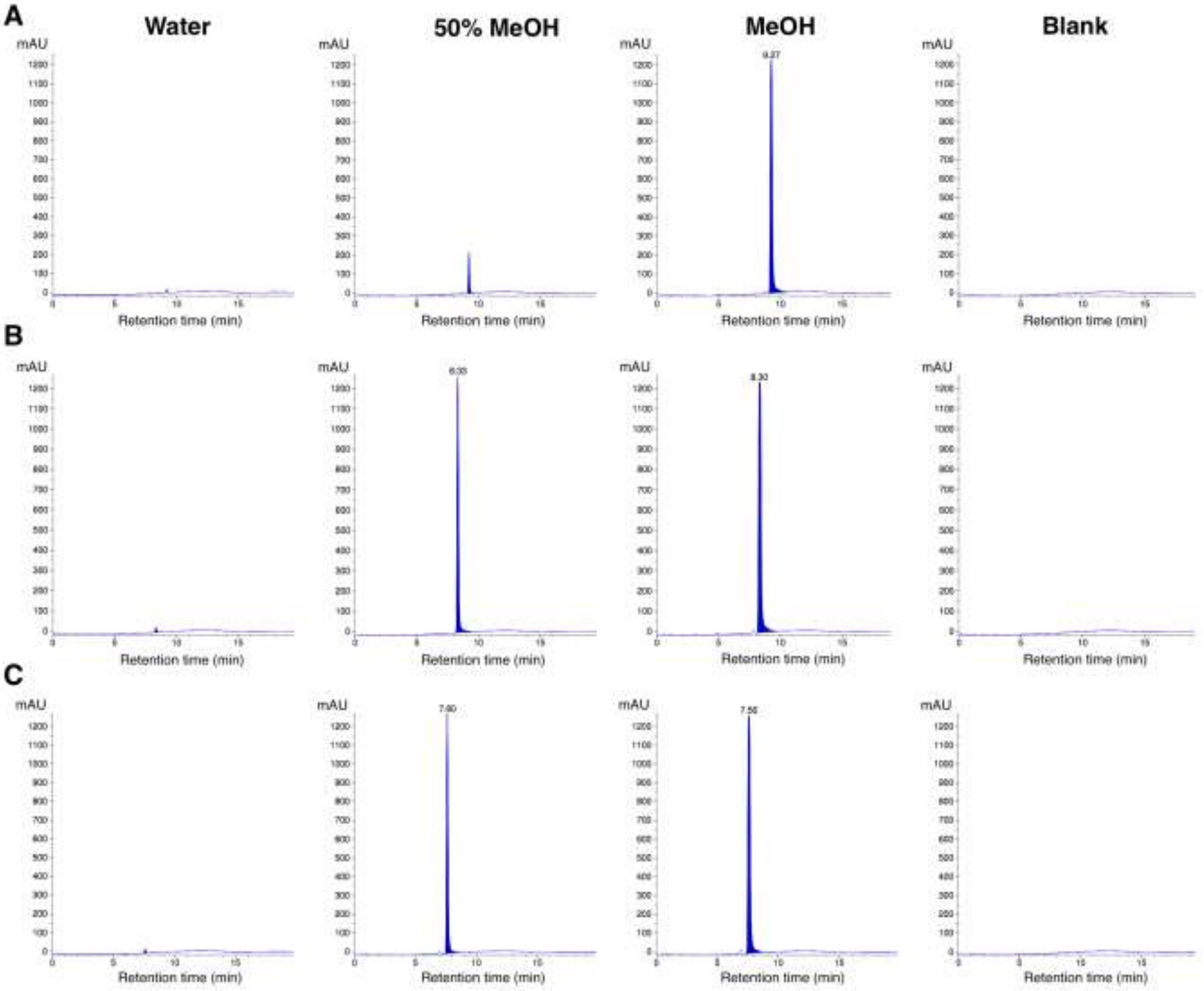

Figure S2. HPLC/ESI-MS chromatograms of compounds NTZ-1091 (A), NTZ-1441 (B), and NTZ-1471 (C) in pure water, 50% methanol, methanol (MeOH). A blank sample was used for each experiment.

Parallel Artificial Membrane Permeability Assay (PAMPA)

Determination of blood-brain barrier (BBB) permeability was performed using the PAMPA Explorer kit (Pion, Inc.). Stock solutions of tested and standard compounds were prepared in DMSO (10 mM and 50 mM for theophylline and lidocaine). The corresponding stock solutions were diluted to a final concentration of 10 µM and 50 µM (5.0 µL/well, 1% v/v final DMSO concentration) with pH 7.4 Prisma HT buffer solution in deionized water (Pion) using a deep-well plate. The first row of this plate was filled with DMSO. For the preparation of pH-dependent PAMPA experiments, the pH of Prisma solution was adjusted to pH 3.0 or 6.2 by adding aliquots of 0.5 M NaOH. The resulting solution (200 µL) was added to each well of the donor plate (n = 6). The polyvinylidene fluoride filter membrane (PVDF, 0.45 µM) on the acceptor plate was coated with 5 µL/well of the GIT-0 lipid (Pion) formulation and placed on the top of the donor plate to form a "sandwich" plate. Then, 200 µL of brain sink buffer (BSB, Pion) was added to each well of the acceptor plate. The "sandwich" plate was incubated in a Good-Box (Pion) at 37 °C for 4 h with stirring at 60 (ABL t.u.). After that, 150 µL/well of each well of the "sandwich" plates were transferred into the corresponding UV read acceptor and donor plates. A blank UV read 96-well plate with 150 µL/well of each buffer solution was prepared. An Epoch microplate reader was used to obtain the UV-vis spectra (250–500 nm) of the solutions in the blank, acceptor, and donor plates. The P_e and –logP_e values for each compound were processed using the PAMPA Explorer software v.3.8 (Pion).

Experimental data for PAMPA-BBB assays

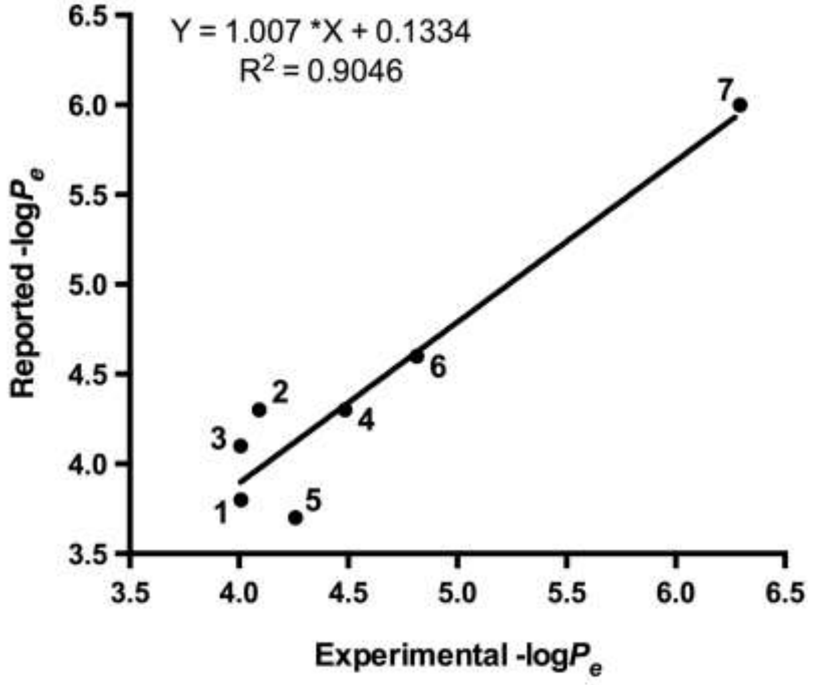

Figure S3. Correlation between reported and experimental permeability values (expressed as –logP_e) of standard drugs used for validation of the PAMPA-BBB assay.

Table S2. Permeability data of standard drugs used for validation of the PAMPA-BBB assay.

**Compound**	**Reported**[a]	**Experiment**		**CNS+/– Prediction**[b,c]
	–logP_e	P_e (× 10^{-6} cm/s)	–logP_e	
Verapamil HCl (**1**)	3.8 ± 0.25	98.0 ± 10.3	4.01 ± 0.05	CNS+
Quinidine HCl (**2**)	4.3 ± 0.25	83.4 ± 20.9	4.09 ± 0.11	CNS+
Propranolol HCl (**3**)	4.1 ± 0.25	98.7 ± 9.96	4.01 ± 0.04	CNS+
Lidocaine (**4**)	4.3 ± 0.25	32.7 ± 0.06	4.49 ± 0.00	CNS+
Progesterone (**5**)	3.7 ± 0.25	55.2 ± 0.02	4.26 ± 0.00	CNS+
Corticosterone (**6**)	4.6 ± 0.25	15.5 ± 0.35	4.81 ± 0.01	CNS±
Theophylline (**7**)	> 6.0 ± 0.25	0.51 ± 0.01	6.29 ± 0.01	CNS–

[a] Ref. 6. [b] Ref. 7. [c] CNS+/– prediction: CNS+ indicates high blood-brain barrier (BBB) permeation predicted, CNS± middle BBB permeation predicted, CNS– low BBB permeation predicted.

Metal binding studies

Interaction with Fe^{2+} and Fe^{3+} ions was determined by measuring the UV-visible absorbance spectra of the respective compound in the absence and in the presence either of $FeCl_3$ or $FeSO_4$ (both from Alfa Aesar). Deferiprone (Santa Cruz Biotechnology, Inc.) was used as positive control. The tested compounds and reference deferiprone were solubilized in DMSO (1.5 mL) and diluted to 50 µM with Ampuwa water-for-injection (Fresenius Kabi). The $FeSO_4$ and $FeCl_3$ samples (10 mM stocks in DMSO) were prepared and protected from light to avoid photo-induced reactions. The UV-visible spectra of the tested compounds were recorded before and after addition of 40 µL either of $FeCl_3$ or $FeSO_4$ at room temperature. The resulting solutions were investigated in time-dependent experiments by recording the UV-visible spectra after additional 30 min, 1, 3, 15, and 24 h incubation at room temperature. Control experiments were performed with 118 µM solutions either of $FeCl_3$ or $FeSO_4$ as well as with 50 µM of compounds alone in 50% DMSO. The UV-visible spectra were recorded on a Jasco V-570 UV-Vis/NIR spectrophotometer (Jasco Analytical Instruments, Inc.) in 1.0 cm quartz cells in the interval of 200–700 nm.

4. References

1. A. Holt, Sharman DF, Baker GB, et al. A continuous spectrophotometric assay for monoamine oxidase and related enzymes in tissue homogenates. Anal Biochem 1997;244:384–92.
2. Tzvetkov NT. Substituted indazole or indole derivatives as in vitro MAO-B inhibitors. 2014;WO2014/107771.
3. Tzvetkov NT, Hinz S, Küppers P, Gastreich M, et al. Indazole- and indole-5-carboxamides: Selective and reversible monoamine oxidase B inhibitors with subnanomolar potency. J Med Chem 2014; 57:6679–703.
4. Tzvetkov NT, Stammler H-G, Neumann B, et al. Crystal structures, binding interactions, and ADME evaluation of brain penetrant N-substituted indazole-5-carboxamides as subnanomolar, selective monoamine oxidase B and dual MAO-A/B inhibitors. Eur J Med Chem 2017;127:470–92.
5. Tzvetkov NT, Neumann B, Stammler H-G, Antonov L. A simple approach to multifunctionalized N1-alkylated 7-amino-6-azaindole derivatives using their *in situ* stabilized tautomer form. Tetrahedron 2016;72:6455–66.
6. Instruction manual for PAMPA Explorer permeability analyzer, Version 4.16, Pion, Inc. 2013.
7. Di L, Kerns EH, Fan K, McConnell OJ, Carter GT. High throughput artificial membrane permeability assay for blood-brain barrier. Eur J Med Chem 2003;38:223-232.
